# Response assessment after induction chemotherapy for head and neck squamous cell carcinoma: From physical examination to modern imaging techniques and beyond

**DOI:** 10.1002/hed.24883

**Published:** 2017-08-17

**Authors:** Remco de Bree, Gregory T. Wolf, Bart de Keizer, Iain J. Nixon, Dana M. Hartl, Arlene A. Forastiere, Missak Haigentz, Alessandra Rinaldo, Juan P. Rodrigo, Nabil F. Saba, Carlos Suárez, Jan B. Vermorken, Alfio Ferlito

**Affiliations:** ^1^ Department of Head and Neck Surgical Oncology UMC Utrecht Cancer Center, University Medical Center Utrecht Utrecht The Netherlands; ^2^ Department of Otolaryngology‐Head and Neck Surgery University of Michigan Health System Ann Arbor Michigan; ^3^ Department of Radiology and Nuclear Medicine University Medical Center Utrecht Utrecht The Netherlands; ^4^ Ear, Nose, and Throat Department, NHS Lothian Edinburgh UK; ^5^ Department of Otolaryngology ‐ Head and Neck Surgery Institut Gustave Roussy Villejuif Cedex France; ^6^ Laboratoire de Phonétique et de Phonologie Sorbonne Nouvelle Paris France; ^7^ Department of Oncology The Sidney Kimmel Comprehensive Cancer Center, Johns Hopkins University School of Medicine Baltimore Maryland; ^8^ Department of Medicine Division of Oncology, Albert Einstein College of Medicine, Montefiore Medical Center Bronx New York; ^9^ University of Udine School of Medicine Udine Italy; ^10^ Instituto Universitario de Oncología del Principado de Asturias University of Oviedo Oviedo Spain; ^11^ Department of Otolaryngology Hospital Universitario Central de Asturias Oviedo Spain; ^12^ Department of Hematology and Medical Oncology The Winship Cancer Institute of Emory University Atlanta Georgia; ^13^ Fundación de Investigación e Innovación Biosanitaria del Principado de Asturias Oviedo Spain; ^14^ Department of Medical Oncology Antwerp University Hospital Edegem Belgium; ^15^ Coordinator of the International Head and Neck Scientific Group

**Keywords:** fluorodeoxyglucose‐positron emission tomography (FDG‐PET), head and neck squamous cell carcinoma, induction chemotherapy, response assessment

## Abstract

Significant correlations between the response to induction chemotherapy and success of subsequent radiotherapy have been reported and suggest that the response to induction chemotherapy is able to predict a response to radiotherapy. Therefore, induction chemotherapy may be used to tailor the treatment plan to the individual patient with head and neck cancer: following the planned subsequent (chemo)radiation schedule, planning a radiation dose boost, or reassessing the modality of treatment (eg, upfront surgery). Findings from reported trials suggest room for improvement in clinical response assessment after induction chemotherapy, but an optimal method has yet to be identified. Historically, indices of treatment efficacy in solid tumors have been based solely on systematic assessment of tumor size. However, functional imaging (eg, fluorodeoxyglucose‐positron emission tomography (FDG‐PET) potentially provides an earlier indication of response to treatment than conventional imaging techniques. More advanced imaging techniques are still in an exploratory phase and are not ready for use in clinical practice.

## 1 INTRODUCTION

The achievement of complete tumor regressions after systemic chemotherapy has been a hallmark of progress in the medical management of solid malignancies. Before the early 1970s, the role of chemotherapy for patients with head and neck cancer was largely limited to palliation of incurable disease. The observations of frequent and significant tumor regressions after chemotherapy alone in previously untreated patients led to the introduction of chemotherapy before surgery or radiation in potentially curable patients in expectation of tumor responses that might permit a reduction in conventional treatment modalities and provide the rationale for subsequent use of chemotherapy as an adjuvant after treatment.^1^ Small studies of chemotherapy alone for laryngeal cancer have reported high rates of complete and durable responses, but the evidence level for chemotherapy alone is low.^2^, ^3^ Thus, the early development of chemotherapy regimens for head and neck cancer uniquely focused on the use of systemic chemotherapy as induction treatment before local treatment modalities. Since that time, the use of induction chemotherapy in the management of locally advanced head and neck squamous cell carcinoma (HNSCC) has grown. Understanding the effects of induction chemotherapy on the biology of the tumor before delivery of definitive treatment (ie, [chemo]radiation or surgery) is paramount to provide as much information as possible in order to tailor the treatment plan to the individual patient: following the planned subsequent (chemo)radiation schedule, planning a radiation dose boost or reassessing the modality of treatment.

Induction chemotherapy, also known as neoadjuvant chemotherapy, has been investigated as a strategy to shrink or downstage locoregionally advanced head and neck cancers, increase organ preservation rates, and/or reduce the risk of locoregional and/or distant recurrences.^4^ The largest meta‐analysis (Meta‐Analyses of Chemotherapy in Head and Neck Cancer) studying the effect of chemotherapy (adjuvant, neoadjuvant, or concomitant) on overall and event‐free survival included 87 trials and 16,485 patients. Induction chemotherapy reduced the risk of distant metastases with a hazard ratio of 0.73.^5^ A more recent meta‐analysis of 14 trials and 2099 patients found no significant difference in overall survival, disease‐free survival, or locoregional recurrence between previously untreated patients and patients with resectable nonmetastatic HNSCC treated with induction chemotherapy followed by locoregional treatment (surgery and/or radiotherapy with or without concomitant chemotherapy) compared with those with locoregional treatment only.^6^ This discrepancy is difficult to explain, but may possibly be due to a difference in primary tumor sites and stages between these different meta‐analyses.^7^ Significant correlations between the response to induction chemotherapy and success of subsequent radiotherapy have been reported and suggest that the response to induction chemotherapy is able to predict a response to radiotherapy.^8^, ^9^, ^10^, ^11^, ^12^ It has been consistently demonstrated in nearly every trial of induction chemotherapy that the survival of responding patients is superior to that of nonresponding patients, suggesting that chemotherapy response is one of the strongest and most reliable prognostic indicators.

The differing clinical responses to induction chemotherapy could lead to different outcomes of (chemo)radiotherapy, with good response leading to high rates of locoregional control by nonsurgical treatment and poor response leading to low rates of locoregional control. Therefore, induction chemotherapy may be used to select patients with resectable HNSCC for organ preservation by (chemo)radiotherapy. It has been well‐recognized that in patients with laryngeal and hypopharyngeal cancer who respond to induction chemotherapy, followed by (chemo)radiotherapy, instead of radical surgery, organ preservation can be achieved without a negative impact on overall or disease‐free survival.^6^, ^9^ For other head and neck tumor sites, there is no conclusive evidence that induction chemotherapy offers the benefit of organ preservation.^6^ Using induction chemotherapy for deintensification of radiotherapy, particularly in human papillomavirus‐associated oropharyngeal carcinoma, is currently being investigated.^13^

In order to assess response to induction chemotherapy without the need for pathological assessment of resected surgical specimen, there is great interest in surrogate metrics for histopathological response. In other tumors (eg, osteosarcoma, locally advanced breast cancer, and esophageal cancers), which are treated by neoadjuvant chemotherapy and/or radiotherapy followed by radical surgery, histopathological examination of the surgical specimen reveals the histologic response to the neoadjuvant treatment. However, if surgical resection is not planned, alternative methods of assessment are needed.

Historically, indices of treatment efficacy in solid tumors have been based solely on systematic assessment of tumor size. Changes in tumor size, particularly complete clinical regression after treatment and the speed of tumor response are often, but not invariably, related to treatment outcome.^14^

In contemporary practice, conventional contrast‐enhanced CT and MRI scans provide the mainstay of imaging for treatment response assessment. Both rely on tumor morphology to evaluate disease, whereas functional imaging, such as positron emission tomography (PET) and diffusion weighted (DW)‐MRI provide complementary information about the underlying tumor biology, such as metabolic activity and cellularity. Changes in tumor metabolism tend to occur early in the course of therapy and, therefore, precede reduction in tumor size.

Therefore, functional imaging potentially provides an earlier indication of response to treatment than conventional imaging techniques. This not only can act as a prognostic indicator but, in addition, may allow for adaptation of definitive treatment planning at a time when this is still feasible. In particular, changes in 18‐fluorodeoxyglucose (FDG) uptake (determined by standardized uptake values [SUVs]) and microscopic water motion (determined by apparent diffusion coefficient [ADC]) are potentially useful for assessment of treatment response. Other techniques include dynamic contrast‐enhanced and perfusion CT and MRI. Optimal timing and interpretation criteria for use of functional imaging in daily practice have yet to be developed.

A variety of approaches for measuring response rate have been developed, including the World Health Organization (WHO) criteria (1979),^15^ the Response Evaluation Criteria in Solid Tumors (RECIST) (2000),^16^ the RECIST 1.1 (2009),^17^ the European Organization for Research and Treatment of Cancer (EORTC) criteria for PET (1999),^18^ the National Cancer Institute guidelines (2006),^19^ and the PET Response Criteria in Solid Tumors (PERCIST) (2009).^20^ Because of variability in measurements and techniques, clinically useful absolute change values reflecting tumor response are lacking. These various classifications divide intrinsically continuous data into bins, losing statistical power in favor of ease of nomenclature, and convenience in clinical practice.

Rapid assessment of treatment effect may allow clinicians to shift patients away from ineffective to effective therapies at an earlier stage (response‐adaptive or risk‐adaptive treatment). Such an approach is an attractive possibility in the drive toward personalized care. Early assessment of therapeutic efficacy is a key issue in considering the potential benefit of up‐front surgery or of treatment escalation (eg, radiation dose boost) in a nonresponder or avoidance of the unnecessary toxicity and costs of ineffective treatments. It is important to realize that a complete metabolic response after induction chemotherapy does not always represent sufficient log cell kill to translate into durable local control, and cure is then achieved by subsequent definitive therapy. That said, the frequent observation of pathological complete responses with chemotherapy alone in HNSCC has been exploited experimentally in small numbers of very highly selected patients demonstrating potential for long‐term disease‐free survival.^21^

### 1.1 Response assessment in randomized clinical trials

Randomized clinical trials are considered by many to be the most reliable form of scientific evidence in the hierarchy of evidence that influences healthcare policy and practice. This is because randomized clinical trials help to reduce spurious causality and bias. Results of randomized clinical trials may be combined into systematic reviews and meta‐analyses, which are increasingly used in the pursuit of evidence‐based medicine. Recently, a meta‐analysis on induction chemotherapy in patients with resectable HNSCC was performed by Ma et al.^6^ From this meta‐analysis, all full articles were selected for review of the response assessment of induction chemotherapy. Response criteria, technique (physical examination and/or imaging), and effect of response assessment are summarized in Table 1.^2^, ^9^, ^10^, ^12^, ^22^, ^23^, ^24^, ^25^, ^26^, ^27^, ^28^, ^29^, ^30^, ^31^, ^32^, ^33^, ^34^, ^35^, ^36^ In the majority of studies, response to induction chemotherapy was assessed by clinical examination, sometimes combined with CT. However, the utilization of endoscopy for objective tumor evaluation has not been fully validated.^16^, ^37^ The majority of studies used the WHO criteria for response assessment. Unfortunately, in most studies, details of clinical assessment for tumor regression were not specified.

**Table 1:** Response assessment of induction chemotherapy in randomized clinical trials

Study	Tumors	Arms			No. of patients	Induction chemotherapy	Cycles	Criteria	Diagnostic technique	Result	Consequence of response
Head and Neck Contracts Program^22^ (1987)	Resectable stage III/IV Oral cavity, hypopharynx, larynx	IC + S + RT IC + S + RT + S S + RT			146 155 161	Cisplatin Bleomycin	1	WHO	Clinical^a^	CR 8% PR 40%	No
Schuller et al^23^ (1988)	Stage III/IV HNSCC	IC + S + RT S + RT			82 76	Cisplatin Methotrexate Bleomycin Vincristine	3	WHO?	Clinical^a^ Pathological	CR 19% PR 51% CR 21%	No
Jortay et al^24^ (1990)	T2/3 piriform sinus	IC + S + RT S + RT			89 98	Vincristine Bleomycin Methotrexate	1	NA	Macroscopic Microscopic	No tumor shrinkage No histopathologic changes	No
VA group^2^ (1991)	Stage III/IV larynx	IC + S + RT	IC + RT S		166 166	Cisplatin 5‐fluorouracil	2	WHO (without confirmation ≥4 wk)	Clinical^a^ Biopsy of primary tumor area	CR 31% PR 54% CR 64%	Third cycle for responders RT for responders and S for nonresponders
Richard et al^25^ (1991)	T2‐4 oral cavity / oropharynx	IC + S ± RT S ± RT			112 110	Vincristine Bleomycin	12 d (intra‐arterial)	WHO (without confirmation ≥4 wk) CR: no living tumor cells PR: islets of living tumor cells NR: no modification of tumor cells	Clinical^a^ Histopathology of surgical specimen	Oral cavity: CR + PR 48% Oropharynx: CR + PR 41% Oral cavity: CR + PR 39% Oropharynx: CR + PR 35%	No
Paccagnella et al^26^ (1994)	Stage III/IV oral cavity / oropharynx/ hypopharynx/ paranasal sinus	IC + RT ± S RT ± S			118 119	Cisplatin 5‐fluorouracil	1‐4	Reevaluation after each cycle CR: total disappearance PR: ≥50% decrease in tumor volume	NA	CR 31% PR 49%	Additional cycle (maximum total 4)
Volling et al^27^ (1994)	T2/3oral cavity / oropharynx/ hypopharynx	IC + S + RT	IC + RT S		49 47	Carboplatin 5‐fluorouracil	1	WHO	Endoscopy and clinical evaluation	CR 44% PR 18%	Additional cycle (maximum total 3) RT for responders and S for nonresponders
Maipang et al^28^ (1995)	Stage III/IV resectable HNSCC	IC + S + RT S + RT			30 24	Cisplatin Methotrexate Bleomycin	2	WHO (without confirmation ≥4 wk)	Clinical or radiological^a^ Histopathology of surgical specimen	CR 30% PR 43% CR 23% PR 53%	No
Lefebvre et al^9^ (1996)	T2‐4 piriform sinus	IC + S + RT	IC + RT S + RT		97 94	Cisplatin 5‐fluorouracil	2	WHO (with for CR also mandatory complete recovery of larynx mobility)	Endoscopic evaluation (CT recommended)	CR 54% PR 32%	Additional cycle (maximum total 3) RT for responders and S + RT for nonresponders
Lewin et al^29^ (1997)	Mainly advanced HNSCC	IC + RT ± S RT ± S			215 208	Cisplatin 5‐fluorouracil	3	No evaluation	N/A	N/A	N/A
Richard et al^30^ (1998)	T3 larynx	IC + S + RT	IC + RT S		36 32	Cisplatin 5‐fluorouracil	2	>80% tumor regression	Direct laryngoscopy	Response 40%	Additional cycle (maximum total 3) RT for responders and S + RT for nonresponders
Kohno et al^31^ (2000)	Stage III/IV oral cavity / pharynx	IC + S	IC + RT S		13 11	Cisplatin Etoposide Mitomycin C	1	WHO	Clinical or radiological^a^	CR 31% PR 23%	Additional cycle (maximum total 2) RT for responders and S for nonresponders
Domenge et al^32^ (2000)	T2‐4 oropharynx	IC + S and/or RT S and/or RT			157 161	Cisplatin 5‐fluorouracil	1	WHO?	Clinical CT (after third cycle)^a^	CR 20% PR 36%	After first: additional cycle unless tumor progression ≥25 After second: additional cycle if tumor regression
Licitra et al^33^ (2003)	T2‐4 oral cavity	IC + S S			98 97	Cisplatin 5‐fluorouracil	2	WHO	Clinical^a^ Pathological	CR 33% PR 49% CR 27% PR 18%	Additional cycle (maximum total 3) –
Urba et al^12^ (2006)	Stage III/IV larynx	IC +	C + RT S	C S	73 19	Cis/carboplatin 5‐fluorouracil	1	WHO	Clinical^a^	CR + PR 75%	Concurrent chemoradiation for responders Surgery for nonresponders
Vermorken et al^34^ (2007)	Stage III/IV unresectable HNSCC	IC + RT			177 181	Docetaxel Cisplatin 5‐fluorouracil Cisplatin 5‐fluorouracil	1	WHO (without confirmation ≥4 wk)	Clinical		Additional cycle (maximum total 4) unless progressive disease
Lefebvre et al^10^ (2009)	T3‐4 larynx T2‐4 hypopharynx	Alternating C + RT IC C+ RT S + RT			224 226	Cisplatin 5‐fluorouracil	2	CR: complete disappearance of all macroscopic disease, with complete recovery of larynx mobility PR larynx: substantial regression of tumor volume, with complete disappearance of bulging valleculae, bulging of hypothyroid membrane, deep invasion of preepiglottic space, and at least partial recovery of larynx mobility PR hypopharynx: substantial regression of tumor volume, with at least partial recovery of larynx mobility	CT / MRI Endoscopy under general anesthesia	CR + PR 85%	Additional cycle (maximum total 2) RT for responders and S + RT for nonresponders
Lorch et al^35^ (2011)	Stage III/IV HNSCC	IC + CRT			255 246	Docetaxel Cisplatin 5‐fluorouracil Cisplatin 5‐fluorouracil	3	No evaluation	N/A	N/A	N/A
Lefebvre et al^36^ (2013)	Stage III/IV larynx / hypopharynx	IC	C + RT Ctx + RT S		60 56 23	Docetaxel Cisplatin 5‐fluorouracil	3	≥50% regression of primary tumor volume or recovered larynx mobility	CT / MRI Endoscopy under general anesthesia	85%	CRT for responders and S + RT for nonresponders

Abbreviations: CR, complete response; CRT, chemoradiotherapy; Ctx, cetuximab; HNSCC, head and neck squamous cell carcinoma; IC, induction chemotherapy; NA, not available; N/A, not applicable; NR, nonresponders; PR, partial response; RT, radiotherapy; S, surgery; WHO, World Health Organization.

a Not further defined / specified.

Some studies show that there is room for improvement in response assessment in categories that currently include complete regression, partial regression, stable disease, and progressive disease. In a study by the Southwest Oncology Group, the rate of clinical complete response (definition and diagnostic techniques not reported) was 19%, whereas the rate of pathological complete response (after induction chemotherapy, all patients underwent surgery) was 13%, suggesting that the clinical assessment used at that time (1980‐1985) was not able to detect all residual disease.^23^

In the final report of the Head and Neck Contracts Program,^22^ in which a single cycle of cisplatin and bleomycin induction chemotherapy was used, a false‐positive rate for histologic complete response was 82%; of the 22 patients with clinical complete response, 18 still had microscopic tumor evident in the surgical specimen. In contrast, 6 of 114 patients (5%) with clinical partial responses had no evidence of cancer in the resected primary tumor.^22^ The EORTC study (1978‐1984) in 97 patients with oral and oropharyngeal cancer also noted a discrepancy between clinical and histopathological regression after induction chemotherapy. Of the 6 patients with clinical complete regression, only 4 patients had pathological complete regression. Of the 46 patients with 50% or more clinical regression, only 31 patients (67%) had a pathological regression, defined as disappearance of living tumor cells (complete response) or persistence of islets of living tumor cells (partial response). Finally, of 48 patients with clinical regression of <50%, 3 patients (6%) were found to have a pathological complete regression.^25^

Maipang et al^28^ reported that in 3 of the 9 patients (33%) with clinical or radiological complete response after 2 courses of induction chemotherapy, tumor was still detected histologically.^28^ In the Veterans Affairs study (started in 1985) on advanced laryngeal cancer, a difference in clinical and pathological assessment results were found; pathologically confirmed complete regression was found in 88% of the patients with clinical complete response and 45% of those with partial response.^8^

In a study by Zhong et al,^38^ 222 patients with advanced stage oral squamous cell carcinoma were randomized between induction chemotherapy (2 cycles of docetaxel, cisplatin, and 5‐fluorouracil) followed by radical surgery and postoperative radiotherapy (range 54‐66 Gy) versus up‐front radical surgery and postoperative radiotherapy. Of 124 patients who received induction chemotherapy, 8.1% were considered to have had a clinical complete response but 13.4% achieved a pathologic complete response. Clinical tumor response was determined by clinical evaluation and imaging studies (performed at baseline and 2 weeks after cycle 2 of induction chemotherapy). The imaging studies were not further specified. Responses were characterized according to the RECIST criteria.^38^

The reported findings suggest room for improvement in response assessment after induction chemotherapy, but an optimal method has yet to be identified.

### 1.2 Morphological response assessment

Imaging at baseline and after 1 or 2 cycles of (induction) chemotherapy can be performed to estimate whether the treatment is effective in that specific tumor and patient. Contrast‐enhanced CT and MRI provide the mainstay of imaging for response assessment in head and neck cancer. The proposed methods to assess treatment response by the WHO criteria include determining the bidimensional measurements of tumors, whereas for RECIST/RECIST 1.1 only unidimensional measurements are used.^16^, ^17^ According to the WHO criteria, for a clinically complete response, no tumor is visible, and for a partial response, tumor is visible but a reduction of >50% of the product of 2 perpendicular diameters is observed, which is confirmed after an interval of at least 4 weeks.^15^ The major reference for justifying a 50% decrease as a criterion for tumor response was based on an experiment in which experienced oncologists had to assess solid wooden spheres placed on a soft mattress and covered with a layer of rubber foam by palpation. Because of measurement errors, the assessed sizes of identical spheres differed by at least 25% in 25% of the measurements and by at least 50% in only 6.8% of the measurements, which was considered acceptable. Thus, if a reduction of 25% in the product of the perpendicular diameters of the “tumors” was considered a response, an unacceptable high false tumor reduction occurred 25% of the time. However, when a 50% threshold was applied, the error fell to an acceptable 7% false‐positive rate.^39^, ^40^

The RECIST criteria, developed by the National Cancer Institute and the EORTC, define response as a 30% decrease in the largest diameter of the tumor. For a spherical lesion, this measure is equivalent to a 50% decrease in the product of 2 diameters (as used in the WHO criteria). Using the RECIST criteria, changes (for at least 4 weeks) are categorized as complete response, partial response, stable disease, or progressive disease. Measurable lesions (used for assessment of response) are defined based on longest diameters, because in smaller lesions the risk of changes by chance is higher. A good concordance was found between response assessment using the RECIST and the WHO criteria for the 4 bins of response in the same patients recruited in 14 different trials. The most precise estimates are achieved when the same imaging technique is used and the same reader assesses the baseline and follow‐up evaluations; more misclassifications and variance in response are noted with different readers. Tumor size is clearly an important parameter.^16^ Due to the irregular 3D shapes of many head and neck tumors, particularly for the oral cavity, the maxilla, and the larynx, the RECIST criteria may not be sensitive for predicting response after chemotherapy, as found by Patil et al^41^ in a small study showing a low correlation between the RECIST response and response on pathological examination.

Traditionally, the morphologic response to therapy has been performed with 2D measurements of size. Advances in CT and MRI techniques and software technology have led to considerable refinements in the accuracy of tumor size measurements facilitating tumor volume measurements. Baghi et al^42^ found a significant difference in tumor volume before and after 3 cycles of induction chemotherapy (docetaxel, cisplatin, and 5‐fluorouracil) in 50 patients with HNSCC. In 78 patients with laryngeal cancer treated with definitive radiation, Issa et al^43^ found that CT scans estimated pretreatment tumor volumes (both primary tumor and composite volumes including nodes) were highly prognostic of success, but that this prognostic value was absent after a single cycle of induction chemotherapy, suggesting that tumor volume assessment after induction chemotherapy is not of prognostic significance. However, further research on the values of volume measurements for clinical response evaluations is warranted. For clinical trials, morphological measurements according to the RECIST 1.1 are recommended (Table 2).^17^

**Table 2:** Response Evaluation Criteria in Solid Tumors criteria

RECIST 1.1	
Target lesion	
Measurable lesions	Longest diameter of tumors or metastasis ≥1.0 cm
	Short axis of lymph node metastasis ≥1.0 cm
Nonmeasurable	Longest diameter <10 mm or pathological lymph nodes with ≥10 to <15 mm short axis)
	Leptomeningeal disease, ascites, pleural or pericardial effusion, inflammatory breast disease, lymphangitic involvement of skin or lung, bone metastasis without soft tissue component
Measurements	Up to 5 lesions, with a maximum of 2 lesions per organ

Abbreviation: RECIST, Response Evaluation Criteria in Solid Tumors.

**Table 3:** Positron Emission Tomography Response Criteria in Solid Tumors criteria

PERCIST 1.0	
Target lesion requirements and selection at baseline	SUVpeak measurement of the hottest lesion (known areas of iatrogenic or benign uptake should not be selected [eg, Waldeyers ring], even when such a lesion has the highest SUVpeak) SUVpeak ≥1.5 times SUVmean liver + 2 SD SUVmean liver In case of extensive liver metastases, SUVpeak ≥2.0 times SUVmean aorta + 2 SD SUVmean aorta It should be reported when no target lesion can be selected because they are below the minimum threshold
Follow‐up lesion selection	SUVpeak measurement of the hottest lesion (may not be the hottest tumor at baseline)
Response measurement and reporting	Reporting of percentage of change in tumor metabolism with notation of number of weeks since treatment start = 100 × [SUVpeak follow‐up target lesion – SUVpeak baseline target lesion] / SUVpeak baseline target lesion
Response categories	
Complete metabolic response Partial metabolic response Stable metabolic disease Progressive metabolic disease	SUVpeak < SUVmean liver and indistinguishable from surrounding background ≥30% decrease of SUVpeak follow‐up target lesion and: ‐ At least 0.8 SUV units decrease of SUVpeak follow‐up target lesion compared to baseline ‐ No new FDG‐avid lesions in a pattern typical of cancer ‐ No increase in size > 30% of target lesion ‐ No increase in size or SUVpeak of >30% in nontarget lesion Increase or decrease of SUVpeak follow‐up lesion <30% Increase of SUVpeak >30% and at least 0.8 SUV units New FDG‐avid lesion(s) in a typical pattern of metastasis

Abbreviations: FDG, fluorodeoxyglucose; SUV, standardized uptake value; SUVmean, standardized uptake value mean; SUVpeak, standardized uptake value peak.

Morphologic measurements are most often used, but have limited value in response assessment after induction chemotherapy to individualize further treatment.

### 1.3 Functional response assessment

Conventional CT and MRI scans rely on morphology to evaluate disease. In contrast, functional imaging, such as PET, DW MRI, dynamic contrast‐enhanced (DCE) MRI, and other advanced functional imaging techniques provide complementary information on the underlying biology. This information includes metabolic activity, cellularity, vascularity, and oxygenation, all of which are potential mediators of chemotherapy and radiation resistance. The reduction in metabolic signal, as depicted on functional imaging, can significantly exceed reductions in morphological volume as defined on CT or MRI.^44^ A minimum of 10 days delay between a chemotherapy cycle and FDG‐PET scanning permits bypassing of the chemotherapeutic effect and transient fluctuations of FDG‐PET that may occur early after treatment (stunting or flare of tumor uptake). This “metabolic flare” is a transient increase in FDG uptake and is thought to consist of 2 effects: an increased metabolism due to cellular stress and an influx of FDG due to damaged cellular membranes.^45^

In several small studies, early therapeutic response on FDG‐PET and DW‐MRI after 2 cycles of induction chemotherapy in patients with advanced‐stage HNSCC seems to be a predictive factor for recurrence‐free survival after subsequent chemoradiation.^46^

### 1.4 Fluorodeoxyglucose‐positron emission tomography

Although a range of factors have been associated with ^18^F‐FDG uptake, there seems to be a rather strong relationship between FDG uptake and cancer cell number. Therefore, it is reasonable to expect that decreases in tumor FDG uptake would be seen with a loss of viable cancer cells.

Although a completely negative PET scan at the end of therapy typically suggests a good prognosis, it does not necessarily correspond to a complete absence of cancer cells, as FDG‐PET is unable to discriminate between minimal tumor burden and no tumor burden. Because FDG uptake is usually not absent in patients who respond well to treatment, prognostic stratification between high and low FDG uptake after or during treatment using absolute cutoff values or cutoff thresholds for percentage decline have been advocated. Metabolic activity and changes due to treatment can be assessed in various ways: qualitative or quantitative; binary (yes or no response); classified (several groups) or continuous (giving varying degrees of response); in the most metabolic active region or the entire tumor volume; in only the primary tumor, the maximal number of lesions or all lesions; and from the same lesion or the most intense lesion (not necessarily the same as the most intense lesion on the other scan).

For early (subtle) changes in tumor uptake before the ultimate treatment effect is complete, quantification may be more desirable than qualitative scoring. Response does likely represent a continuum of intensities of uptake. Because PET is intrinsically a quantitative imaging method, quantitative measurement of early treatment‐induced changes is an attractive tool for measuring subclinical response and more complete changes. More than 30 different ways to assess tumor response by PET quantitatively have been reported, but SUVs are the most widely applied, generally correlating well with more complex analytic approaches. The SUV is a widely used metric for assessing tissue accumulation of tracers. The SUV can be normalized to total body mass, lean body mass, or body surface area. Although these SUV normalization approaches will give different absolute change in SUVs with effective treatment and different absolute amount of change to be significantly different from a previous scan, the percentage changes with treatment will be comparable in a single patient with a stable weight and identical patient preparation and scan protocol.^20^

A wide variety of region of interest (ROI) selection metrics has been used: manually defined ROI (tumor delineation), isocontour ROIs based on a fixed percentage of the maximal pixel in tumor, fixed SUV threshold, or a background‐level threshold and fixed dimension. The most frequently used SUV metric is the SUV obtained from the pixel with the highest uptake within the tumor (SUVmax). Another SUV metric is standardized uptake value peak (SUVpeak), which is defined as the average SUV within a small, fixed‐size ROI (a 1 cm^3^ volume spheric ROI) centered on a high‐uptake part of the tumor.^20^

The SUV reproducibility, which is important in clinical practice, is mainly dependent on the ROI and lesion size. Small lesions may have low uptake of FDG due to partial volume effect. The SUVmax can easily be measured using modern commercial workstations and is most resistant to partial volume effect in small tumors, but is highly dependent on the pixel size. The SUVpeak in a small volume of greatest metabolic activity in the tumor is less subject to variance than is a small, single pixel SUVmax. Because SUVs of small lesions are more susceptible to measurement faults, tumor sizes should be noted and should be 2 cm or larger in diameter for accurate measurement, although smaller lesions of sufficient FDG uptake, including those not well seen anatomically, can be assessed. Generally, lesions must be clearly visible and both large enough and hot enough to evaluate changes in SUV.^20^ Standardization, as proposed in the United States^19^ and Europe,^36^ is essential to achieve reproducible SUVs.

A variety of methods has been used to determine the change in SUVs associated with treatment. Absolute SUV and percentage decline in SUV can both be used to assess treatment response. The ratio of SUV is less dependent on ROI choice than absolute SUV determinations and is, therefore, preferred.^47^ Moreover, using absolute SUV decline in multicenter studies and comparing between reported studies may be difficult due to inadequate standardization of SUV determination. An SUV decline of 30%‐35% is usually associated with a good outcome. However, the decline warranted for achievement of treatment goal may be dependent on tumor type, treatment performed, and time interval after treatment.^47^

In patients with multiple lesions, several strategies to assess response to therapy by SUV decline have been described: (1) assessment of SUVmax (the single, most intense area in the primary tumor (not necessarily the same area), which is considered to coincide with the worst‐case biologic behavior of malignancy) decline of the primary tumor only, because changes in SUV of the primary tumor seem to predict the outcomes in metastases quite accurately; and (2) the smallest percentage decline in SUVpeak of a lesion as representative, with the rationale that the lesion with the worst response would determine survival.^20^

The medically relevant cutoff value for an SUV decline to optimally represent response and predict outcome may differ on the basis of disease, the timing after treatment, the treatment itself, and the treatment goal. Early during treatment, lower cutoff values may be used than following completion of treatment. In addition, for induction chemotherapy, this cutoff value may be lower as further treatment is foreseen. This cutoff value can be used for response‐adaptive treatment (eg, concurrent [chemo]radiotherapy with eventual additional cycles of induction chemotherapy for responders or surgery with or without postoperative radiotherapy in nonresponders). Decisions to deny probably ineffective therapy depend on alternative therapeutic options available and on the risk, costs, and perceived benefits of available treatment options. In the case of false‐positive findings, when induction chemotherapy is followed by radiotherapy instead of defaulting to surgical resection, tumor relapse is more likely to occur. This may then require salvage surgery with a higher risk of postoperative complications. With regard to predicting further response to subsequent radiotherapy, it is not always essential to achieve histopathological complete response after induction chemotherapy.

The delay in translating PET as response metric from research to clinical practice is probably due to the variability in study performance (imaging protocol) and the lack of uniformly practiced response metrics for PET. Standardized approaches to the performance of PET and to machine calibrations have been articulated.^19^, ^47^ Qualitative and quantitative approaches for PET treatment response assessment have been postulated.^16^, ^20^

In the RECIST 1.1, FDG‐PET scanning may only be incorporated to complement CT scanning in assessment of progression.^17^ The only exception to this is in the use of FDG‐PET imaging as an adjunct to determination of progression, as described later in this guideline. Recently, Hyun et al^48^ reported on a simplified guide to PERCIST 1.0, which describes in detail methods for controlling the quality of FDG‐PET imaging conditions to ensure the comparability of PET images from different time points to allow quantitative expression of changes in PET measurements for an assessment of overall treatment response in PET studies. The PERCIST uses the SUVpeak corrected for lean body mass (SULpeak) and defines criteria for measurable lesions. In short, responses are categorized in: (1) complete metabolic response: complete resolution of FDG uptake; (2) partial metabolic response: a decrease of ≥30% and of at least 0.8 SUL units between the most intense evaluable lesion at baseline and follow‐up (not necessarily the same lesion); (3) stable disease: an increase or decrease in SULpeak of <30%; (4) progressive disease: an increase ≥30% and an increase of at least 0.8 SUL units in target lesion or development of a new lesion (Table 3).^20^, ^48^ For functional response assessment using FDG‐PET in clinical trials, the PERCIST criteria are recommended.

**Table 4:** Clinical studies using fluorodeoxyglucose‐positron emission tomography for response assessment after induction chemotherapy

Study	No. of patients	Induction chemotherapy	Second scan (time after completion of chemotherapy)	Parameter	Lesion	Measure	Cutoff point	Reference standard	Accuracy
Brun et al^50^ (2002)	10	1 cycle cisplatin, 5‐FU	0‐5 d	Metabolic rate SUVpeak	Primary tumor	Absolute	Median	Follow‐up (median 3.3 yr)	Local control < mean 96% ≥ mean 62% (*P* = .007) Local control < mean 91% ≥ mean 68% (*P* = .07)
Chepeha et al^37^ (2009)	12	1 cycle cis‐/carboplatin, 5‐FU	3 wk	SUVmax (3 × 3 pixel)	Primary tumor	Visual estimation of decrease	50%	Endoscopy	Substantial agreement
Kikuchi et al^53^ (2011)	15	1 cycle S‐1 and CDGP	Mean 20.5 (14‐31) d	SUVmax	Primary tumor and largest lymph node	Absolute Decrease	3.5 55.5.%	<10% viable tumor in tumor bed in surgical specimen	Sens 71% Spec 89% PPV 71% NPV 89% Sens 86% Spec 95% PPV 86% NPV 95%
Semrau et al^58^ (2015)	47	1 cycle docetaxel, cisplatin	3 wk	SUVmax	Primary tumor	Decrease	20%	>30% reduction in superficial tumor extension	Sens 97% Spec 56% PPV 90% NPV 83%
Wong et al^59^ (2016)	20	2 cycles docetaxel, cisplatin, 5‐FU	After first cycle	TLG	Primary tumor	Decrease	60%	Follow‐up 3 mo after completion of chemoradiation	Sens 73% Spec 80%
Gavid et al^57^ (2015)	21	2‐3 cycles docetaxel, cisplatin, 5‐FU	After first cycle	SUVmax	Primary tumor	Decrease	30%	≥70% response with endoscopy after end of induction chemotherapy	Sens 69% Spec 63% PPV 75% NPV 90%
Yoon et al^55^ (2011)	21	2 cycles S‐1 and cisplatin	2‐4 wk	SUVmax	Primary tumor	Absolute Decrease	4.8 65%	RECIST 2 mo after completion chemoradiation	Sens 94% Spec 100% PPV 100% NPV 80% Sens 88% Spec 100% PPV 100% NPV 67%
Powell et al^44^ (2013)	9	2 cyclescisplatin, 5‐FU	NA	–	Primary tumor Lymph node	Visual residual avidity	Yes / no	Follow‐up and neck dissection	Sens NA Spec 89% PPV NA NPV 100% Sens 100% Spec 88% PPV 50% NPV 89%
Dalsaso et al^49^ (2000)	19	2‐3 cycles paclitaxel and cisplatin		SUVmean	Primary tumor	Decrease	–	4 biopsies from 4 separate sites within pretreatment tumor area	Pathologic complete responders mean reduction 82%, nonresponders mean reduction 35% (*P* = .01)
McCollum et al^51^ (2004)	26	3 cycles cisplatin, 5‐FU +/‐ docetaxel	NA	‐	Primary tumor	Visual estimation of residual tumor	Yes / no	Biopsy of primary tumor site	Sens 100% Spec 65% PPV 27% NPV 100%
Abgral et al^46^ (2012)	15	3 cycles docetaxel, cisplatin, 5‐FU	Mean 15.8 ± 4.9 d after second cycle	SUVmax	Primary tumor	Decrease	EORTC criteria; metabolic response: SUVmax decrease >25%	1‐y event‐free survival (mean follow‐up 14.3 ± 6.6 mo)	Metabolic responders 0%, nonresponders 27% survived 1 y
Yu et al^56^ (2014)	28	3 cycles docetaxel, cisplatin, 5‐FU	2‐3 wk	MTV TLG	Primary tumor	Decrease	42% 55%	Event‐free survival	Sens 67% Spec 90% Sens 63% Spec 90%

Abbreviations: 5‐FU, 5‐fluorouracil; CDGP, XXX; EORTC, European Organisation for Research and Treatment of Cancer; MTV, metabolic tumor volume; NA, not available; NPV, negative predictive value; PPV, positive predictive value; RECIST, Response Evaluation Criteria in Solid Tumors; Sens, sensitivity; Spec, specificity; SUVmax, standardized uptake value maximum; SUVpeak, standardized uptake value peak; TLG, total lesion glycolysis.

### 1.5 Clinical studies

Dalsaso et al^49^ performed CT and FDG‐PET before and after 2 or 3 cycles of paclitaxel and carboplatin in 19 patients with advanced head and neck cancer. A suboptimal reference standard was used: 4 biopsies from 4 separate sites within the tattooed primary tumor area before treatment. A significant difference in mean reduction of SUVmean was found in complete pathologic responders, defined as patients with negative biopsies after chemotherapy (82%) as compared with patients having residual disease (35%). Although no significant difference in mean reduction in tumor volume by CT between these patient groups was observed, a significant correlation between percent reduction of SUVmean and percentage reduction in CT tumor volume after chemotherapy was found.^49^

In patients with locally advanced HNSCC, Brun et al^50^ found that patients who had an SUVpeak of FDG lower than the median value after 1 cycle of chemotherapy or 12‐40 Gy radiotherapy have a higher tumor response and better survival as compared to those with a higher than median SUVpeak. Unfortunately, patients who underwent FDG‐PET after induction chemotherapy were not separately evaluated.^50^

McCollum et al^51^ analyzed the FDG‐PET results of 26 patients with advanced‐stage head and neck cancer after 3 cycles of induction chemotherapy with cisplatin and 5‐fluorouracil with or without docetaxel. When the outcome of histopathological examination of a biopsy from the primary tumor site was used as reference standard, a high sensitivity (100%) and negative predictive value (100%) but a low specificity (65%) and positive predictive value (27%) were found for detecting persistent disease at the primary site. However, the possibility of false‐negative results from biopsy specimen sampling errors could not be ruled out.^51^

The FDG‐PET and CT were compared with endoscopy and biopsy while the patients were under general anesthesia 3 weeks after a single course of induction chemotherapy with cisplatin or carboplatin and 5‐fluorouracil in 12 patients with resectable advanced‐stage oropharyngeal cancer by Chepeha et al.^37^ During endoscopy with the patients under general anesthesia after induction chemotherapy, the percent of residual primary tumor was estimated by the surgeons relative to the tattoo markings made during the pretreatment endoscopy. Endoscopy was used as reference standard and to decide whether to continue with nonsurgical treatment (concomitant chemoradiation followed by adjuvant chemotherapy if response is at least 50%) or subsequent surgery with postoperative radiotherapy (if response is lower than 50%). Although SUVmax values were determined for each tumor, these SUVs were only used as addition to visual estimation of response and not for calculation of SUVmax change. Tumor volumes were assessed on CT. The agreement between PET and endoscopy was substantial and the agreement between CT and endoscopy was fair. They suggest that FDG‐PET is more reliable than CT for predicting tumor response, although the reference standard was not ideal. The authors hypothesize that FDG‐PET can replace endoscopy with biopsies for assessment of tumor response after induction chemotherapy.^37^

Argiris et al^52^ reported on a series of 39 patients with locally advanced head and neck cancer who underwent induction docetaxel, cisplatin, and cetuximab followed by concurrent radiotherapy, cisplatin, and cetuximab and maintenance cetuximab. Response assessment was performed after 3 cycles of this induction protocol by CT, physical examination, and the FDG‐PET portion of PET/CT. Complete response by PET was defined as complete disappearance of FDG activity attributable to malignancy, without regard to the degree of CT response, as assessed on combined PET/CT. Substantial differences in complete response rate as assessed by CT, physical examination, and PET were reported: for primary tumor 48%, 70%, and 58%, and for lymph node metastases 5%, 34%, and 21%, respectively.^52^

Kikuchi et al^53^ evaluated the predictive value of sequential FDG‐PET/CT after 1 cycle of neoadjuvant chemotherapy (the platinum complex CDGP and the oral fluoropyrimidine derivate S‐1) in 16 patients with HNSCC. They used the SUVmax of 15 primary tumors and 11 (largest) lymph nodes and grading of histopathological regression as the reference standard (response: <10% vital tumor in tumor bed; nonresponse 10% or more vital tumor in tumor bed). Although 2 different PET/CT scanners were used, sequential PET/CT scans before and after induction chemotherapy were performed using the same protocol and scanner for each patient. Postchemotherapy SUVmax (cutoff point 3.5) and percentage decline in SUVmax (cutoff point 55.5%) were shown to predict histopathological responses with a sensitivity of 71% and 86%, a specificity of 89% and 95%, a positive predictive value of 71% and 86%, and a negative predictive value of 89% and 95%, respectively. The MRI findings based on longest diameter before and after chemotherapy were not able to predict histopathological response in these same patients.^53^ In a later study, Kikuchi et al^54^ used the same SUVmax decrease threshold of 55.5% for defining responders and nonresponders to induction chemotherapy by FDG‐PET/CT evaluation in comparison to the RECIST with MRI evaluation. Only nonresponders revealed by FDG‐PET/CT were significantly linked to poor local tumor control rate and disease‐specific survival (hazard ratio 4.9).^54^

Yoon et al^55^ evaluated the efficacy of FDG‐PET after 2 cycles of induction chemotherapy (S‐1 and cisplatin) in 21 patients with advanced‐stage head and neck cancer who achieved partial response to predict clinical outcome after concurrent chemoradiation. Patients who attained a complete response (according to RECIST) after concurrent chemoradiation showed a significantly higher decrease in SUVmax compared with patients who failed to attain a complete response. An SUVmax of at least 4.8 on FDG‐PET after induction chemotherapy and a decrease from baseline of at least 65% in SUVmax after induction chemotherapy predicted complete response after concurrent chemoradiation and progression‐free and overall survival.^55^

The potential of FDG‐PET/CT after 2 (or 3) cycles of docetaxel, cisplatin, and 5‐fluorouracil to predict disease‐free survival in 15 patients with locally advanced HNSCC treated by induction chemotherapy preceding concomitant chemoradiation was evaluated by Abgral et al.^46^ Metabolic response was assessed by the measurement criteria of the EORTC. The 1‐year disease‐free survival of metabolic responders, defined as at least 25% decrease (between baseline and after 2 cycles of induction chemotherapy) of SUVmax, was statistically significantly better than nonresponders (100% vs 20%; *P* = .0014).^46^

A greater reduction in FDG‐avid volume and, hence, metabolic signal than in reduction of volume on conventional imaging (CT and MRI) after induction chemotherapy was observed by Powell et al.^44^

The FDG volumetric imaging parameters to assess response to induction chemotherapy were used by Yu et al^56^ in 28 patients with advanced‐stage HNSCC who underwent 3 cycles of TPF chemotherapy (docetaxel, cisplatin, and 5‐fluorouracil) followed by chemoradiation. Different parameters for metabolic tumor volume and total lesion glycolysis were evaluated. A reduction of 42% of metabolic tumor volume and 55% of total lesion glycolysis were predictive of progression‐free survival after subsequent chemoradiation with a sensitivity of 67% and 63% and a specificity of 90% and 90%, respectively.^56^

In a preliminary study, Gavid et al^57^ assessed the correlation between reduction in SUVmax and in metabolic tumor volume (measured from isocontours of SUV = 2.5) after a first cycle of induction TPF chemotherapy (docetaxel, cisplatin, and 5‐fluorouracil) and clinical response, as assessed by endoscopy with taking of biopsies after 2 or 3 cycles of induction chemotherapy in 21 patients with advanced‐stage HNSCC. Using this suboptimal reference standard, patients with advanced‐stage HNSCC with ≥70% tumor reduction on endoscopy and negative biopsies were considered to be responders and continued with chemoradiation, whereas nonresponders underwent surgery. Responders showed a significantly greater mean SUVmax reduction between PET/CT examinations pretreatment and after 1 cycle of chemotherapy. Responders tended to show greater reduction in hypermetabolic volume than nonresponders.^57^

Semrau et al^58^ performed response assessment using FDG‐PET/CT and endoscopic evaluation after a single cycle of induction chemotherapy using docetaxel and cisplatin or carboplatin in 47 patients with advanced‐stage HNSCC. Responders achieving a ≥30% decrease in endoscopic tumor size and a ≥20% decrease in SUVmax proceeded to chemoradiation and nonresponders to surgery. In 89% of these patients, metabolic and clinical responses were similar. Using this strategy of selecting patients for chemoradiation or surgery, a local control rate of 86% was obtained.^58^

Assessment by PET/CT and DW‐MRI after the first cycle of induction chemotherapy (docetaxel, cisplatin, and 5‐fluorouracil) in 20 patients with advanced‐stage HNSCC who received 2 cycles of induction chemotherapy followed by concomitant chemoradiation was reported by Wong et al.^59^ Responders, defined as patients without persistent disease at response assessment at 3 months after completion of chemoradiation with MRI, PET/CT, and clinical examination, showed a significantly greater reduction in metabolic tumor volume and total glycolysis both measured for ROI with uptake of ≥40% of SUVmax and with SUV ≥3.5.^59^

Recently, a systematic review on the effectiveness of FDG‐PET/CT for evaluating early response to induction chemotherapy in HNSCC was performed. Seven studies including a total of 207 patients with advanced‐stage HNSCC were included. The authors concluded that a meta‐analysis was not possible because the selected studies were heterogeneous concerning response criteria, reference standards, chemotherapy strategy, and endpoints. However, 6 of 7 studies concluded that FDG‐PET allowed early evaluation response to induction chemotherapy and predicted survival outcomes.^60^

The studies cited above demonstrate the potential of FDG‐PET to assess response to induction chemotherapy in order to select patients for treatment adaptation (ie, concomitant chemoradiation or surgery). Unfortunately, different parameters have been used for response assessment. Moreover, SUV cutoffs identified in (single center) studies involving a specific set of patients may not be applicable to other centers with different equipment, patient populations, chemotherapy regimens, and clinical imaging protocols. Reporting the SUV changes as figures and not only as the PERCIST criteria would be helpful to assess the most useful cutoff value, as otherwise the advantages of the continuous output of PET data are lost through forced categorization.

If induction chemotherapy is used to select patients with resectable HNSCC for organ preservation by (chemo)radiotherapy, response assessment would preferably be performed after only 1 cycle in order to avoid further unnecessary treatment with its associated burden, toxicity, and morbidity. In Table 4, studies using FDG‐PET for response assessment after induction chemotherapy are ordered according to the timing of the assessment. Unfortunately, due to aforementioned heterogeneity, only the conclusion can be made that even after one cycle FDG‐PET is very promising for this purpose.

## 2 MORE RECENT ADVANCES IN IMAGING

### 2.1 Diffusion‐weighted MRI

Diffusion‐weighted DW‐MRI, which provides maps of microscopic water motion within biologic tissues, offers a simplistic approach (as compared to CT perfusion and DCE‐MRI) to physiologic changes within the tumor after treatment. Higher cellularity (eg, malignant tumor) is generally associated with more restricted diffusion (lower ADC values).

Because ADC measurements are dependent on a high number of adjustments, which differ between scanners and protocols, results from studies are not generally applicable across different institutes, hampering its implementation.^61^ Changes in ADC values are probably less dependent on DW‐MRI scanners and protocols than absolute ADC values.

Cytotoxic therapy triggers tumor cell death, leading to reduced density with a subsequent increase in ADC after treatment. Berrak et al^62^ evaluated the potential of DW‐MRI in monitoring the treatment response of the largest metastatic cervical lymph node in patients with HNSCC undergoing cisplatin‐based induction chemotherapy. Each patient underwent an MRI on the same 2 scanners used. Changes in nodal volume, signal intensity on T2, and ADC were not different for complete and partial responders at different clinical endpoints. Although no difference in changes in nodal volume and signal intensity on T2 were found between survivors and those who died of HNSCC, a significant difference in percentage change in ADC between those patient groups was observed.^62^ In the previously mentioned study of Wong et al,^59^ a trend was observed for a higher ADC on DW‐MRI after 1 cycle of induction chemotherapy in responders compared with nonresponders of induction chemotherapy followed by concomitant chemoradiation.^59^

Conventional DW‐MRI cannot separate perfusion and true diffusion‐related effect. Intravoxel incoherent motion (IVIM) imaging is characterized by 3 parameters: pure diffusion coefficient; microvascular volume fraction; and perfusion‐related incoherent microcirculation. The IVIM‐derived parameters may characterize the actual status of diffusion in tumors more accurately then conventional DW because it provides both perfusion and true diffusion‐related measurements. In a recent study by Guo et al,^63^ IVIM measurements were performed before and after 2 cycles of paclitaxel and cisplatin induction chemotherapy in 28 patients with advanced‐stage hypopharyngeal carcinoma. Response was classified according to RECIST 3 weeks after the second cycle of induction chemotherapy by conventional MRI. The posttreatment ADC and pure diffusion coefficient were significantly higher in responders than in nonresponders, whereas perfusion‐related incoherent microcirculation was significantly lower in responders and microvascular volume fraction was not significantly different. Changes (between pretreatment and 3 weeks after induction chemotherapy) of ADC, pure diffusion coefficient, and perfusion‐related incoherent microcirculation were significantly higher in responders, but microvascular volume fraction was not.^63^

The DW‐MRI is a promising technique for response assessment, but further research using a standardized protocol is needed for eventual implementation in clinical practice.

### 2.2 CT and MRI perfusion

A number of methods have been developed for the measurements of tissue perfusion using CT and MRI. These methods can generally be grouped under 2 classes: compartmental analysis and deconvolution‐based methods. Perfusion studies are obtained by monitoring a standard iodinated contrast of gadolinium bolus through a vascular bed.

Deconvolution‐based CT perfusion is a fast imaging technique, which can assess physiologic parameters, such as blood flow, blood volume, mean transit time, and capillary permeability surface area product, and provides data that can be useful in the detection and characterization of tumor. Significant perfusion differences of blood flow, blood volume, mean transit time, and capillary permeability have been found in untreated HNSCC compared with adjacent normal tissue.^64^ CT perfusion has been proposed as a new, possibly superior evaluation of tumor response. After intravenous injection of a bolus iodinated contrast agent, tissue and vessel attenuation changes can be observed during the first pass of the agent by dynamic image acquisition at a given anatomic level. Time‐density curves can be constructed for observer‐defined ROI. Within limits of assumptions, tissue perfusion can be estimated based on observed density changes. The time course of the iodine concentration is a measure of the regional perfusion, and this concentration is linearly correlated to tissue density, as seen on CT. Several algorithms can be used to measure perfusion with CT. Gandhi et al^65^ examined whether these CT perfusion parameters correlate with response to induction chemotherapy as assessed by endoscopy with the patient under general anesthesia. In 9 patients with advanced head and neck cancer, reduction in blood volume by >20% on CT perfusion 3 weeks after 1 cycle of induction chemotherapy (cisplatin and 5‐fluorouracil) showed substantial agreement with clinical response (≥50% reduction in tumor volume), as assessed with endoscopy. The agreement among decreased (≥20%) blood flow, decreased (≥20%) capillary permeability, and increased (≥20%) mean transit time and clinical response was fair. Based on these results, the authors hypothesized that CT perfusion parameters could potentially replace invasive diagnostic procedures with the patient under general anesthesia as a predictor of tumor response.^65^

Petralia et al^66^ found a correlation between a decrease in both blood flow and blood volume on perfusion CT and tumor volume reduction in 20 patients with advanced‐stage head and neck cancer after 2 cycles of cisplatin and 5‐fluorouracil as induction chemotherapy.

In DCE‐MRI, several parameters can be computed pixel‐wise: transfer constant (K^trans^), the volume of extravascular extracellular space per unit volume of tissue (Ve), the initial (60s) area under the gadolinium curve (IAUGC60) and the enhancing fraction. Powell et al.^44^ reported a significant fall of the transfer constant K^trans^ and IAUGC60 after chemotherapy.

These advanced perfusion imaging techniques for response assessment are still in an exploratory phase and not ready for use in clinical practice.

### 2.3 Future development of response assessment

The clinical utility of imaging after induction chemotherapy but before subsequent locoregional therapy is based on the ability to predict clinical response and survival after sequential definitive therapy (ie, concurrent [chemo]radiation or surgery). It is not always essential to achieve complete response after induction chemotherapy, because subsequent definitive (chemo)radiation may eradicate residual disease. Rough classification systems for tumor response have been used for decades because precise techniques were not or later not yet widely available. However, more recent morphological and functional imaging techniques may allow for more reliable reporting on changes during or after treatment. Therefore, individual figures can be used in reporting response assessment and categories can be made based on optimal cutoff values. Because new treatment paradigms and new imaging modalities and techniques require continued reevaluation of response assessment tools, recently, the RECIST working group proposed organ‐specific modifications. However, these are not yet defined for head and neck cancer.^67^

Although the WHO and the RECIST criteria are historically focused on a reliable assessment of any response after induction chemotherapy, new quantitative functional imaging techniques will determine a cutoff value for optimal prediction of response after subsequent chemoradiation. These cutoff values will be dependent on alternative treatment options, available treatment modifications, and the opinions of patients and their clinicians. However, when new techniques are evaluated for their potential role in determination of response to induction chemotherapy, an initial correlation between imaging parameters and response have to be investigated.

Several studies suggest that functional imaging techniques show potential in determining response to induction chemotherapy when compared to morphological radiological or clinical assessments. However, the wide variety of methodologies and endpoints reported limit the conclusions that can be drawn at this stage. Nevertheless, functional imaging holds promise for more personalized treatment using induction chemotherapy to select patients with HNSCC for definitive therapy.

### 2.4 Use of biomarkers for response assessment

A major goal of response assessment to induction chemotherapy is proper selection of patients for subsequent management based on the biologic response of the tumor to initial cytotoxic chemotherapy in anticipation of improved survival and/or organ preservation. However, if surrogate biomarkers could predict the response to chemotherapy, treatment selection for definitive therapy could be improved, toxicities reduced, redundant treatment avoided, and perhaps other biologic methods to monitor response could be developed that would guide changes in therapy.^68^, ^69^ In general, it seems consistent that tumor or molecular characteristics that reflect rapid tumor growth or high cellular proliferation tend to correlate with responses to induction chemotherapy, whereas lack of aggressive growth, proliferation, or invasiveness tend to predict better responses to surgical excision.

### 2.5 Response assessment after induction immunotherapy

Although the focus of this review is response assessment after induction chemotherapy, the immunotherapy of head and neck cancer is the most rapidly developing frontier of treatment and has been stimulated by the approval of several immune checkpoint inhibitors for clinical use in patients with advanced cancers.^70^, ^71^ Further, induction immune modulation is gaining increasing popularity as a means to assess the clinical and immunologic effectiveness of these agents.^72^ Like the development of induction chemotherapy approaches, methods to predict and appropriately assess tumor and immunologic responses after induction immune modulation are needed. However, it is unclear if the clinical or radiologic measures that have been proven useful after induction chemotherapy will be equally useful after immunotherapy because effects of immune‐mediated cytotoxicity tend to evolve more slowly than direct cytotoxic agents and may be accompanied by initial tumor swelling, acute inflammation, or increased functional activity due to influx of tumor‐infiltrating lymphocytes. Appropriate metabolic imaging will likely be more meaningful than anatomic imaging. Considerable effort is underway to define both molecular and immune markers that predict success of immune modulation with the checkpoint inhibitors. Clearly, what has been learned regarding the monitoring of tumor response to induction chemotherapy could have important implications for the development of induction immunotherapy regimens for patients with head and neck cancers. Patients with head and neck cancers will continue to represent an ideal model for future development of induction chemotherapy and immune therapy regimens and associated biomarkers to guide selection of appropriate definitive treatment modalities for more personalized care.

## 3 CONCLUSION

Induction chemotherapy may be used to tailor the treatment plan to the individual patient with head and neck cancer: following the planned subsequent (chemo)radiation schedule, planning a radiation dose boost, or reassessing the modality of treatment (ie, up‐front surgery). For this purpose, reliable response assessment is needed. Response assessment after induction chemotherapy is currently probably most valuable if a choice between an organ‐preservation approach (radiotherapy with or without chemotherapy) and surgery has to be made, particularly for hypopharyngeal and laryngeal cancer. Response assessment using conventional clinical morphological techniques is limited. Functional imaging, in particular using FDG‐PET, is promising, but the introduction in routine clinical practice is limited due to the variability in study performance (imaging protocol) and the lack of uniformly practiced response metrics for PET. Research on other functional imaging techniques for response assessment is scarce and these techniques are still in an exploratory phase. Surrogate biomarkers, which predict the response to chemotherapy and may be used to select definitive therapy with less toxicity, are under investigation. To allow comparison of clinical trial results and development of guidelines for the use of induction chemotherapy, it is essential that for response assessment radiological measurements are performed according to current guidelines using the RECIST 1.1 (if only morphological imaging is performed) and the PERCIST 1.0 criteria on accredited PET/CT scanners. Whereas the RECIST criteria are historically focused on a reliable assessment of any response after induction chemotherapy, new quantitative (functional imaging) techniques will attempt to predict response after subsequent chemoradiation using cutoff values. Optimal cutoff values can only be determined if trial results are reported as continuous data and not only in categories of response.
